# Maltodextrin-Based Carbohydrate Oral Rinsing and Exercise Performance: Systematic Review and Meta-Analysis

**DOI:** 10.1007/s40279-022-01658-3

**Published:** 2022-03-03

**Authors:** Claudia Hartley, Amelia Carr, Steven J. Bowe, Wender L. P. Bredie, Russell S. J. Keast

**Affiliations:** 1 CASS Food Research Centre, Deakin University, 221 Burwood Highway, Burwood, VIC 3125 Australia; 2 Centre for Sport Research, Deakin University, 221 Burwood Highway, Burwood, VIC 3125 Australia; 3 Deakin Biostatistics Unit, Faculty of Health, Deakin University, 221 Burwood Highway, Burwood, VIC 3125 Australia; 4 Department of Food Science, University of Copenhagen, Rolighedsvej 26, 1958 Frederiksberg, Denmark

## Abstract

**Background:**

Carbohydrates are an important fuel for optimal exercise performance during moderate- and high-intensity exercise; however, carbohydrate ingestion during high-intensity exercise may cause gastrointestinal upset. A carbohydrate oral rinse is an alternative method to improve exercise performance in moderate- to high-intensity exercise with a duration of 30–75 min. This is the first systematic review and meta-analysis to comprehensively examine the isolated effect of maltodextrin-based rinsing on exercise performance.

**Objective:**

The objective of this review was to establish the effect of a maltodextrin-based carbohydrate oral rinse on exercise performance across various modes of exercise. Furthermore, a secondary objective was to determine the effects of moderators [(1) participant characteristics; (2) oral rinse protocols; (3) exercise protocol (i.e. cycling, running etc.) and (4) fasting] on exercise performance while using a maltodextrin-based, carbohydrate oral rinse.

**Methods:**

Five databases (MEDLINE, PsycINFO, Embase, SPORTDiscus and Global Health) were systematically searched for articles up to March 2021 and screened using Covidence (a systematic review management tool). A random effects robust meta-analysis and subgroup analyses were performed using Stata Statistical Software: Release 16.

**Results:**

Thirty-five articles met the inclusion criteria and were included in the systematic review; 34 of these articles were included in the meta-analysis. When using a conventional meta-analytic approach, overall, a carbohydrate oral rinse improved exercise performance in comparison with a placebo (SMD = 0.15, 95% CI 0.04, 0.27; *p* = 0.01). Furthermore, when implementing an adjusted, conservative, random effects meta-regression model using robust variance estimation, overall, compared with placebo, a carbohydrate oral rinse demonstrated evidence of improving exercise performance with a small effect size (SMD = 0.17, 95% CI − 0.01, 0.34; *p* = 0.051).

**Conclusion:**

This systematic review and meta-analysis demonstrates that a maltodextrin-based carbohydrate oral rinse can improve exercise performance. When comparing the two meta-analytic approaches, although non-significant, the more robust, adjusted, random effects meta-regression model demonstrated some evidence of a maltodextrin-based carbohydrate oral rinse improving exercise performance overall.

**Supplementary Information:**

The online version contains supplementary material available at 10.1007/s40279-022-01658-3.

## Key Points

**Table:** 

According to the robust, meta-regression model, a maltodextrin-based carbohydrate oral rinse shows some evidence of improving exercise performance.
According to a conventional meta-analytic approach, rinsing a maltodextrin-based carbohydrate oral rinse for 10 s with a concentration between 6 and 6.5% is effective at improving exercise performance.

## Introduction

In the 1920s, it was recognised that carbohydrates were a crucial fuel source for exercise [1] with improved exercise capacity and general exercise performance being linked to carbohydrate consumption [2]. In subsequent research, it was demonstrated that a glucose polymer solution improved exercise capacity during cycling time to exhaustion tests compared with a placebo solution [3, 4]. Similar findings indicated that time to fatigue was significantly longer after ingesting a glucose polymer solution and glucose infusion in comparison with a placebo [3]. Currently, it is standard practice for individuals to ingest carbohydrates prior to or during sustained high intensity or endurance exercise [5–8]. Based on literature by Burke et al. [9], the American College of Sports Medicine (ACSM) recommends the total daily intake of 5–7 g per kilogram per day of carbohydrates for a moderate daily exercise programme (i.e. ~ 1 h/day); which can be ingested either prior to exercise, during exercise or in recovery from a previous exercise session [9–11]. The ACSM details further recommendations for acute fuelling strategies for carbohydrate loading, pre-event fuelling and for during brief exercise (< 45 min), sustained high-intensity exercise (i.e. 45–75 min) and endurance exercise (1–2.5 h). During sustained high-intensity exercise, it is currently recommended that small amounts of carbohydrates be ingested (including mouth rinsing) for optimal carbohydrate intake, whereas during endurance exercise, it is recommended that 30–60 g of carbohydrates be ingested per hour [9, 10]. A potential disadvantage associated with carbohydrate ingestion, however, is the possible occurrence of gastrointestinal discomfort [6, 12–15], which can subsequently negatively affect exercise performance [16–18].

An alternative method to utilise carbohydrates during exercise is a carbohydrate oral rinse. Previous research indicates that a maltodextrin rinse comprising of a 6–6.4% maltodextrin-based solution [19–21] during moderate- to high-intensity exercise with a time span ranging from 30 to 75 min can facilitate improvements in exercise [6, 8]. The exact mechanism that facilitates improvements in exercise performance after a carbohydrate oral rinse remains unknown. It is proposed that alterations in exercise performance may be influenced by a ‘Central Governor’ mechanism to maintain homeostasis during exercise [22]. The ‘Central Governor’ is thought to modify power output through the use of afferent signals from peripheral physiological receptors and systems that detect changes in the external and internal environment [22]. Therefore, it could be interpreted that during exercise, the positive central responses to a carbohydrate oral rinse could possibly counteract the negative physical, metabolic and thermal afferent signals [23]. An alternative theory is that improved exercise performance is a result of enhanced brain activation in higher brain regions. It is thought that these higher brain regions link the corresponding cognitive, behavioural and emotional response and the gustatory pathways [24, 25]. Furthermore, these regions have been found to be activated by oral exposure to carbohydrates but not by non-nutritive sweeteners [19, 26, 27], which may assist in explaining the positive effects of carbohydrate rinsing on exercise performance.

Carter et al. [28] first investigated the effects of a carbohydrate oral rinse on performance during a cycling time trial. During the time trial, participants were instructed to complete a certain amount of work (kJ) as quickly as possible. This amount of work was based on a formula including each participant’s maximum power output value (*W*_max_) [28]. During the time trial either a 6.4% (w/v) maltodextrin or water (placebo) sample was rinsed in the mouth for 5 s prior to expectoration. With the carbohydrate oral rinse, performance time was significantly faster (2.9%) in comparison with the water rinse (placebo) [28]. Additionally, improvements in exercise performance after an oral carbohydrate rinse in comparison with a placebo rinse have been found with cycling [29–33], running [34–37] and resistance exercise [38]. In contrast, some studies have reported no significant improvements in exercise performance [39–46]. This lack of significant improvements may be due to the study design (i.e. mode of exercise, concentration and/or composition of the rinse and rinsing duration) or lack of statistical power to detect changes. Due to the inconsistent results in the pool of literature, further analysis is required to investigate if a maltodextrin-based oral rinse does improve exercise performance.

Furthermore, research in this area has also discussed a possible placebo effect in conjunction with carbohydrate oral rinsing. As previous research has demonstrated that placebo effects may have a significant impact on physical performance [47], it is common practice for at least two oral rinses to be trialled: a carbohydrate oral rinse and a placebo oral rinse [28, 29]. To minimise possible placebo effects between the rinsing conditions, previous research has also blinded participants to the composition of the rinses and also to the true objective of the experiment [45].

Previous reviews have focused on the effects of carbohydrate oral rinsing on exercise performance across running and cycling performance [48], cycling performance [49] and sprinting performance [50], and carbohydrate oral rinsing alongside ingestion and loading on exercise performance [51]. However, no reviews have specifically discussed the intricacies of the maltodextrin used in the carbohydrate oral rinse. Maltodextrin is a variable starch-based structure [52] that is a widely used product in foods and food manufacturing [53]. Maltodextrin can vary depending on its physical and chemical properties, which can in turn affect the overall flavour and appearance [54]. Additionally, as maltodextrin can vary widely in terms of structure and origin, these may be important factors to investigate as this variation may impact exercise performance. For example, starches are composed of two types of glucose polymers: amylose and amylopectin [55]. The ratio between amylose and amylopectin can affect the physical properties of starches including their retrogradation tendencies, viscosity and pasting properties [56–58]. Other important structural factors include dextrose equivalent (DE) and degree of polymerisation (DP). For example, a shorter-chain maltodextrin has a higher DE and lower DP and therefore has a sweeter taste in comparison with a longer-chain maltodextrin [54]. The origin of the maltodextrin may also be an important factor to consider as maltodextrin can be made from corn, rice, manioc, oat or potato starch [59]. Depending on the source of the starch, the ratio of amylose to amylopectin changes. For example, high-amylose corn starch has an amylose content of 50–70%, whereas potato, tapioca and wheat starches have an amylose content close to 20% [58]. Without reporting on the type of maltodextrin used in the carbohydrate oral rinse, the information concerning origin and structure is unknown, potentially prohibiting informed observations and mechanistic insights. Furthermore, oral rinse protocol (concentration and duration) is an important factor to investigate as dose response or time/duration response with a carbohydrate oral rinse and exercise performance response may exist. Exercise protocol, fasting and participant characteristics are additional factors that can also vary across the literature and are important to investigate as there may be an optimum level or conditions at which exercise performance can be improved. The primary aim of this systematic review and meta-analysis is to comprehensively examine the isolated effect of maltodextrin-based rinsing on exercise performance. Furthermore, the secondary aim of this review is to investigate the effect of the concentration and composition of the rinse, duration of the rinse and the impact of participant characteristics (i.e. sex), fasting and exercise protocol on exercise performance.

## Methods

The systematic review and meta-analysis was completed according the Cochrane Handbook for Systematic Reviews of Interventions [60] and following the PRISMA (Preferred Reporting Items for Systematic Reviews and Meta-Analyses) statement [61].

### Eligibility Criteria

For this systematic review and meta-analysis, studies were included if they met the following inclusion criteria: (1) investigated the effect of a maltodextrin oral rinse on exercise performance; (2) randomised, blinded counterbalanced or crossover, control or placebo study design; (3) a maltodextrin-based carbohydrate oral rinse with a concentration of no less than 6% being rinsed for a minimum of 5 s (based on existing literature in the area [28, 49]); (4) original research articles and (5) human participants. Studies were excluded if they (1) did not use maltodextrin in the oral rinse; (2) involved any ingestion of the maltodextrin rinse; (3) were not original research articles (i.e. conference abstracts, review articles); (4) were not written in English and (5) did not have sufficient methodological information to allow a check of the inclusion criteria. The search included articles that were published up to and including February 2021.

### Data Sources and Search

Initially, a small test search using the chosen search terms was conducted to determine the efficacy of the search terms. After the test search confirmed the search strategy was effective, the search terms were finalised. Five separate databases (MEDLINE, PsycINFO, Embase, SPORTDiscus and Global Health) were searched and this was performed initially in June 2020 and updated in March 2021. The following terms were used in the search: Carbohydrate *OR* CHO *OR* Carbohydrates *AND* Oral Rinse *OR* Oral Rinsing *OR* Mouth Rinsing *OR* Mouth Rinse *OR* Rinsing *OR* Rinse *OR* Mouth Wash *AND* Exercise Performance *OR* Performance *OR* Exercise *OR* Cycling *OR* Running *OR* Sprinting *OR* Resistance.

**Fig. 1:**
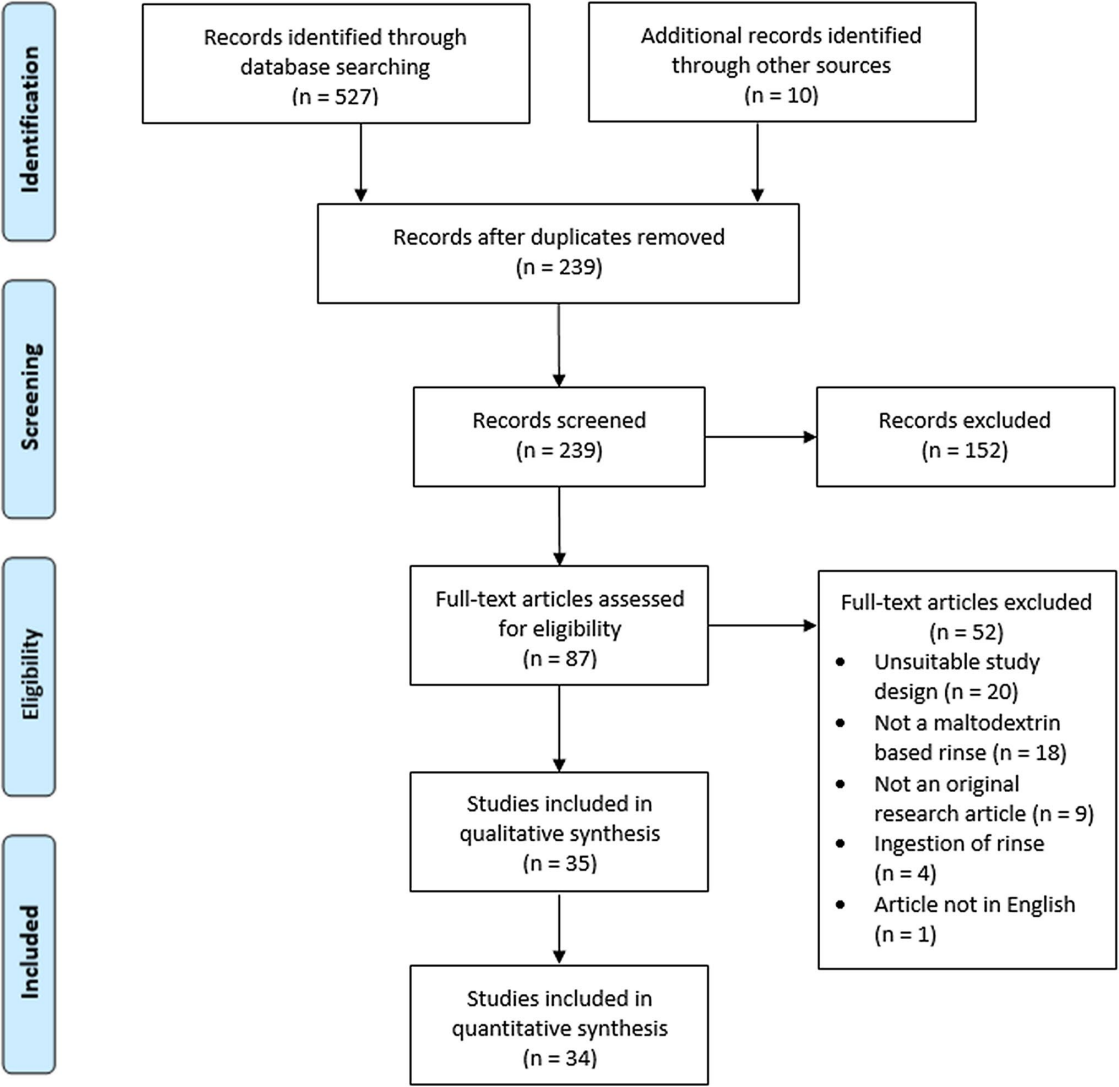
PRISMA flow diagram [62]

This search strategy yielded 527 publications. The hand searching technique, which involved searching reference lists of included studies and review articles for relevant studies, found a further ten studies. From the pool of included studies, 288 duplicates were removed which resulted in 239 studies for screening. After screening, a total of 35 studies were included for data extraction and analysis for the systematic review and of these 35 studies, a total of 34 studies were included in the meta-analysis. No relevant studies were found within grey literature. The process of study selection and screening is summarised in a PRISMA flow diagram (Fig. 1).

### Study Selection and Data Collection

The studies were imported into a systematic review management tool (Covidence, Veritas Health Innovation, Victoria, Australia) to complete the screening process. The titles, abstracts and full texts of the studies were reviewed separately to check for eligibility criteria. The screening process for abstract screening and full-text screening was completed by three separate reviewers. Screening conflicts were resolved through discussion between the three independent reviewers until a conclusion was reached. Data extraction for the included studies focused on (1) title, year of publication, type of publication and (2) methods and design of the study, participants selected (sample size, sex and age of participants), intervention (composition and concentration of oral rinse), type of exercise performed and outcome measures (i.e. distance covered in the trial [m], time to complete the trial [s], power output [W], speed [km/h]). As the majority of studies reported results as mean ± standard deviation, studies that reported mean ± standard error were converted to mean ± standard deviation for consistency. If additional information was required from a study for data extraction, the corresponding author was contacted. On the occasion where this further information was unable to be obtained, that study was excluded from the meta-analysis.

### Quality Assessment

This quality assessment was completed using a modified version of the Quality Criteria Checklist: Primary Research [63] and previously published formatting [64]. One author (C.H.) performed the quality assessment and a second author (R.S.J.K.) independently cross-checked the quality assessment. Any disagreements were discussed and resolved between the two authors.

### Risk of Bias Assessment

A risk of bias assessment was conducted with the studies that were included in the meta-analysis according to the Cochrane Collaboration’s recommendation for systematic reviews [65]. The categories for assessment included (1) random sequence generation (selection bias); (2) blinding of participants and personnel (performance bias); (3) blinding of outcome assessment (detection bias); (4) incomplete outcome data (attrition bias) and (5) selective reporting (reporting bias). Each category was assessed and assigned either a low risk of bias, high risk of bias or unclear risk of bias. One author (C.H.) performed the risk of bias assessment and a second author (R.S.J.K.) independently cross-checked the risk of bias assessment. Any disagreements were discussed and resolved between the two authors.

### Statistical Analysis

The data extraction process for the meta-analysis focused on primary performance-based outcomes (refer to Table 1 for a complete list). Outcomes that were not deemed to be performance based were excluded from the meta-analysis data set. Studies with multiple results for a single performance outcome (i.e. maximal speed: sprint 1, sprint 2, sprint 3 etc.) were collapsed and averaged together prior to the meta-analysis. From the 34 included articles, the data collection process resulted in 58 data points for analysis.

**Table 1:** Studies investigating the effect of a maltodextrin-based carbohydrate oral rinse on exercise performance

References	Exercise protocol	Sample (*n*)	Training status	Fast (h)	Duration of mouth rinse (s)	Rinse composition/concentration (%)	Washout period	Performance outcomes (mean ± SD)	Statistical significance
Andersson et al. [39]	Arm cranking	12 (males)	Physically active	**	5	6.4% Maltodextrin	7 days	Mean distance (km): CHO: 12.3 ± 1.4 vs PLA: 11.9 ± 1.5	No (*p* = 0.164)
Ataide-Silva et al. [29]	Cycling TT	8 (males)	Physically active	Fasted group: 12	10	6.4% Maltodextrin	Min 72 h, Max 7 days	Time (min): CHO: 42.47 ± 1.39 vs PLA: 43.90 ± 1.51	Yes ↑ (*p* = 0.009)
Bailey et al. [68]	Isometric contractions	10 (5 males, 5 females)	Moderate to high activity levels	**	20	6.4% Maltodextrin and artificial sweeteners and colouring	Min 48 h	*Immediately*: motor evoked potential (%): CHO: 18.28 ± 17.61 vs PLA: − 2.19 ± 13.44; *Immediately*: maximal voluntary contraction (N·m) (%): CHO: 7.66 ± 6.10 vs PLA: − 3.24 ± 4.74	Yes ↑ (*p* ≤ 0.05)
								*After 10 min*: motor evoked potential (%): CHO: 31.54 ± 40.38 vs PLA: − 13.41 ± 23.59; *After 10 min*: maximal voluntary contraction (N·m) (%): PLA: 10.18 ± 4.74 vs PLA: − 6.46 ± 7.02	Yes ↑ (*p* ≤ 0.05)
Bastos-Silva et al. [69]	Resistance exercise	12 (males)	Resistance training for 2 + years	**	10	6.4% Maltodextrin and juice	72 h	Repetitions (leg press): CHO: 13.5 ± 4.8 vs PLA 11.5 ± 4.4 vs CON: 12.4 ± 4.4; training load volume (kg) (leg press): CHO: 2006.7 ± 825.2 vs PLA: 1712.5 ± 772.9 vs CON: 1817.1 ± 672.6	No (*p* > 0.05)
								Repetitions (bench press): CHO: 8.2 ± 1.6 vs PLA: 7.1 ± 2.4 vs CON: 6.8 ± 1.8; training load volume (kg) (bench press): CHO: 557.1 ± 155.4 vs PLA: 495.9 ± 206.1 vs CON: 476.1 ± 173.3	Yes, with CHO vs CON only ↑ (*p* ≥ 0.05)
Bavaresco Gambassi et al. [30]	TTE cycling	21 (males)	Physically active, non-cyclists	3	10	6.4% Maltodextrin and lemon flavouring	7 days	Time (min): CHO: 70.9 ± 30.3 vs PLA: 57.4 ± 30.6 vs CON: 43.0 ± 27.5	Yes ↑ (*p* < 0.001)
Bazzucchi et al. [2]	Isometric contractions	18 (Males)	Moderately active	8	10	6.4% Maltodextrin	72 h	Total work (J): CHO: 4249 ± 780.65 vs PLA: 3853 ± 759.43 vs CON: 3729 ± 602.45 vs WAT: 3888 ± 759.43	Yes ↑ (*p* = 0.004)
Beelen et al. [40]	Cycling TT	14 (males)	Competitive cyclists (trained twice per week)	Overnight	5	6.4% Maltodextrin	Min 7 days	Mean time (min): CHO: 68.14 ± 4.27 vs PLA: 67.52 ± 3.74; mean power output (W): CHO: 265 ± 18.71 vs PLA: 266 ± 18.71	No (*p* > 0.05)
Black et al. [70]	Maximum voluntary contractions	13 (6 males, 7 females)	Recreationally active	**	20	8% Maltodextrin and artificial sweetener	48–96 h	Maximal voluntary contraction (N·m): CHO: 230 ± 90 vs PLA: 232 ± 90; voluntary activation (%): CHO: 91.9 ± 2.9 vs PLA: 91.5 ± 3.8	No (*p* > 0.05)
Carter et al. [28]	Cycling TT	9 (7 males, 2 females)	Endurance-trained athletes	4	5	6.4% Maltodextrin	7 days	Mean time (min): CHO: 59.57 ± 4.50 vs PLA: 61.37 ± 4.68; mean power output (W): CHO: 259 ± 48 vs PLA: 252 ± 48	Yes ↑ (*p* ≤ 0.05)
Chambers et al. [19]	Cycling TT	8 (males)	Endurance-trained cyclists	Overnight	~ 10	6.4% Maltodextrin and artificial sweeteners	Min 3 days	Mean time (min): CHO: 62.6 ± 4.7 vs PLA: 64.6 ± 4.9; mean power output (W): CHO: 225 ± 57 vs PLA: 217 ± 55	Yes ↑ (*p* ≤ 0.05)
Cherif et al. [41]	Running sprints	15 (males)	Recreationally active	14 for 3 days	5	10% Maltodextrin	24 h	*Sprint 1:* Maximal power (w/kg): CHO: 14.1 ± 3.8 vs PLA: 13.7 ± 3.6 vs CON: 15.4 ± 4.2; maximal speed (s): CHO: 4.5 ± 0.9 vs PLA: 4.5 ± 0.8 vs CON: 4.7 ± 0.9	No (*p* > 0.05)
								*Sprint 2:* Maximal power (w/kg): CHO: 12.8 ± 3.5 vs PLA: 13.3 ± 3.9 vs CON: 13.1 ± 3.3; maximal speed (s): CHO: 4.3 ± 0.8 vs PLA: 4.3 ± 0.9 vs CON: 4.4 ± 0.8	No (*p* > 0.05)
								*Sprint 3:* Maximal power (w/kg): CHO: 12.6 ± 3.9 vs PLA: 12.2 ± 3.8 vs CON: 12.8 ± 4.3; maximal speed (s): CHO: 4.3 ± 0.9 vs PLA: 4.3 ± 1.0 vs CON: 4.2 ± 1.0	No (*p* > 0.05)
								*Sprint 4:* Maximal power (w/kg): CHO: 15.1 ± 3.7 vs PLA: 13.9 ± 3.7 vs CON: 11.6 ± 3.8; maximal speed (s): CHO: 4.7 ± 0.9 vs PLA: 4.5 ± 1.0 vs CON: 4.1 ± 0.9	No (*p* > 0.05)
								*Sprint 5:* Maximal power (w/kg): CHO: 13.3 ± 3.7 vs PLA: 13.0 ± 4.1 vs CON: 13.6 ± 3.9; maximal speed (s): CHO: 4.4 ± 0.9 vs PLA: 4.3 ± 1.0 vs CON: 4.4 ± 1.0	No (*p* > 0.05)
								*Sprint 6:* Maximal power (w/kg): CHO: 12.1 ± 5.0 vs PLA: 13.1 ± 4.1 vs CON: 13.6 ± 4.44; maximal speed (s): CHO: 4.4 ± 1.0 vs PLA: 4.4 ± 1.0 vs CON: 4.4 ± 1.0	No (*p* > 0.05)
								*Sprint 7:* Maximal power (w/kg): CHO: 12.5 ± 3.9 vs PLA: 15.0 ± 3.4 vs CON: 12.6 ± 3.5; maximal speed (s): CHO: 4.3 ± 1.0 vs PLA: 4.6 ± 0.7 vs CON: 4.3 ± 0.9	No (*p* > 0.05)
								*Sprint 8:* Maximal power (w/kg)**:** CHO: 13.8 ± 3.3 vs PLA: 13.5 ± 3.5 vs CON: 14.0 ± 3.5; maximal speed (s): CHO: 4.4 ± 0.8 vs PLA: 4.4 ± 0.8 vs CON: 4.5 ± 0.8	No (*p* > 0.05)
								*Sprint 9*: Maximal power (w/kg): CHO: 14.1 ± 3.8 vs PLA: 13.4 ± 4.1 vs CON: 1.2 ± 3.5; maximal speed (s): CHO: 4.5 ± 0.8 vs PLA: 4.4 ± 1.0 vs CON: 4.4 ± 0.8	No (*p* > 0.05)
								*Sprint 10:* Maximal power (w/kg): CHO: 12.6 ± 3.7 vs PLA: 12.9 ± 3.6 vs CON: 13.1 ± 3.7; maximal speed (s): CHO: 4.5 ± 0.8 vs PLA: 4.4 ± 1.0 vs CON: 4.4 ± 0.8	No (*p* > 0.05)
Chong et al. [42]	Cycling sprints	14 (males)	Trained cyclists	Overnight	5	6.4% Maltodextrin	Min 7 days	Maximum power output (W): CHO: 1191 ± 130.96 vs WAT: 1203 ± 112.25 vs CON: 1206 ± 145.92	No (*p* > 0.05)
								*0–30 s* Mean power output (W)**:** CHO: 859 ± 78.57 vs WAT: 855 ± 67.35 vs CON: 854 ± 71.09	No (*p* > 0.05)
								*0–10 s* Mean power output (W): CHO: 1001 ± 115.99 vs WAT: 1009 ± 97.28 vs CON: 1006 ± 493.90	No (*p* > 0.05)
								*10–20 s* Mean power output (W): CHO: 877 ± 78.57 vs WAT: 868 ± 71.09 vs CON: 866 ± 71.09	No (*p* > 0.05)
								*20–30 s* Mean power output (W)**:** CHO: 699 ± 59.87 vs WAT: 696 ± 52.38 vs CON: 695 ± 56.12	No (*p* > 0.05)
Clarke et al. [71]	Resistance exercise	15 (males)	Recreationally resistance trained	**	10	6% Maltodextrin and artificial sweetener	Min 2 days	1-Repetition maximum (kg): CHO: 90 ± 17 vs PLA: 86 ± 17 vs CON: 88 ± 18; repetitions (bench press): CHO: 21 ± 5 vs PLA: 22 ± 5 vs CON: 21 ± 4; total exercise volume (number of repetitions × weight lifted [kg]): CHO: 1135 ± 360 vs PLA: 1137 ± 324 vs CON: 1106 ± 335	No (*p* > 0.05)
Clarke et al. [72]	Various exercises	12 (males)	Train 3–4 times per week	Overnight (11)	10	6% Maltodextrin and orange flavouring	7 days	Countermovement jump height (cm): CHO: 39 ± 7 vs PLA: 38 ± 7 vs CON: 36 ± 6; 10-m sprint time (s): CHO: 1.78 ± 0.07 vs PLA: 1.81 ± 0.07 vs CON: 1.85 ± 0.05	Yes ↑ (*p* ≤ 0.05)
								Repetitions (bench press): CHO: 25 ± 3 vs PLA: 24 ± 4 vs CON: 22 ± 4; repetitions (squat): CHO: 31 ± 4 vs PLA: 29 ± 5 vs CON: 26 ± 6	Yes ↑ (*p* ≤ 0.05)
								Isometric mid-thigh pull peak force (N): CHO: 2262 ± 288 vs PLA: 2236 ± 354 vs CON: 2212 ± 321	No (*p* = 0.368)
Clarke et al. [73]	Running TT	15 (9 males, 6 females)	Recreational runners	5	10	6%, 12% Maltodextrin and non-caloric sweetener	7 days	Mean time (min): CHO 6% rinse: 27:05 ± 3:52 vs CHO 12% rinse: 26:47 ± 4.31 vs 0% rinse (PLA): 26:34 ± 4:07	No (*p* > 0.05)
Cramer et al. [74]	Cycling TT	8 (males)	Endurance trained	**	5	6.5% Maltodextrin and lime flavouring	5–7 days	Mean time (min): CHO: 63.9 ± 3.2 vs PLA: 64.3 ± 2.8; Mean power output (W): CHO: 251 ± 23 vs PLA: 242 ± 18	No (*p* > 0.05)
de Oliveira et al. [75]	Running sprints	14 (males)	University futsal players	**	10 & 40	6% Maltodextrin	72 h	Best time (s): CHO 10-s rinse: 7.79 ± 0.19 vs CHO 40-s rinse: 7.71 ± 0.15 vs PLA: 7.77 ± 0.28	No (*p* > 0.05)
								Mean time (s): CHO 10-s rinse: 8.18 ± 0.37 vs CHO 40-s rinse: 8.15 ± 0.62 vs PLA: 8.18 ± 0.21	No (*p* > 0.05)
								Total sprint time (s): CHO 10-s rinse: 49.12 ± 0.21 vs CHO 40-s rinse: 48.92 ± 0.36 vs PLA: 49.02 ± 0.82	No (*p* > 0.05)
Decimoni et al. [38]	Resistance exercise	15 (females)	1–2 years prior strength training experience	8	10	6% Maltodextrin and artificial sweetener	72 h	Total workload volume: CHO: 7.589 ± 1.914 vs PLA: 6.678 ± 1.1741	Yes ↑ (*p* = 0.039)
Deighton et al. [76]	Walking	18 (males)	Recreationally active	Overnight	10	6.4% Maltodextrin and artificial sweetener	~ 7 days	Mean distance difference (m): CHO vs WAT: 163; PLA vs WAT: 134; CHO vs PLA: 29	No (*p* = 0.204)
Dorling and Earnest [43]	Running sprints	8 (males)	Physically active	**	~ 5	6.4% Maltodextrin and artificial sweetener	7–9 days	Mean time (s): CHO: 3.44 ± 0.17 vs PLA: 3.46 ± 0.2; fastest time (s): CHO: 3.37 ± 0.2 vs PLA: 3.38 ± 0.2; mean sprint time (LIST) (s): CHO: 3.54 ± 0.2 vs PLA: 3.52 ± 0.2	No (*p* > 0.05)
Dunkin and Phillips [44]	Resistance exercise	12 (males)	Recreationally resistance trained	1.5	10	18% Maltodextrin and citrus flavouring	2–7 days	1-Repetition maximum: CHO: 91.67 ± 18.77 vs PLA: 90.83 ± 18.27 vs CON: 91.25 ± 19.35	No (*p* = 0.680)
								Repetitions (bench press): CHO: 40.58 ± 5.09 vs PLA: 39.92 ± 4.23 vs CON: 39.67 ± 5.73	No (*p* = 0.677)
								Total exercise volume: CHO: 1478.75 ± 309.24 vs PLA: 1437 ± 263.73 vs CON: 1435.92 ± 338.34	No (*p* = 0.600)
Durkin et al. [77]	Resistance exercise	12 (males)	Resistance trained	2	10	6.4% Maltodextrin and artificial sweetener	**	Total volume workload (kg): CHO: 9354 ± 2051 vs PLA: 8525 ± 1911; repetitions (squat): CHO: 107 ± 26 vs PLA: 92 ± 16	Yes ↑ (*p* ≤ 0.05)
								Repetitions (bench press): CHO: 120 ± 24 vs PLA: 115 ± 22	No (*p* = 0.146)
Fares and Kayser [31]	TTE cycling	13 (males)	Not physically active	Fasted group: overnight	5–10	6.4% Maltodextrin	72–96 h	*Fed:* Mean time (min): CHO: 56.6 ± 12.2 vs PLA: 54.7 ± 11.3; * Fasted:* Mean time (min): CHO: 53.9 ± 12.8 vs PLA: 48.3 ± 15.3	Yes ↑ (*p* ≤ 0.05)
Gam et al. [45]	Cycling TT	10 (males)	Cyclists	**	5	6.4% Maltodextrin	7 days	Time (min): CHO: 65.7 ± 11.07 vs PLA: 69.4 ± 13.81 vs CON: 67.6 ± 12.68	Yes, with CHO vs PLA and CON vs PLA ↑ (*p* ≤ 0.05)
Green et al. [78]	Resistance exercise	36 (18 males, 18 females)	Resistance trained	12	10	6.4% Maltodextrin and Powerade Zero	48–72 h	Repetitions (bench press): CHO: 18.7 ± 0.8 vs PLA: 19.0 ± 0.7 vs WAT: 17.7 ± 0.8	Yes, with CHO and PLA vs WAT ↑ (*p* ≤ 0.05)
Jeffers et al. [46]	Cycling TT	9 (males)	Cyclists and triathletes	4	5	6.4% Maltodextrin	Min 7 days	Mean power output (W): CHO: 248 ± 23 vs PLA: 248 ± 39	No (*p* = 0.997)
Jensen et al. [79]	Isometric knee extension	12 (males)	Actively training (3–4 sessions per week)	Overnight	10	8% Maltodextrin and artificial sweetener	4.6 ± 2.2 days	*Pre-Fatigue:* Peak torque (N.m): CHO: 364.02 ± 43.82 vs PLA: 351.55 ± 37.66	No (*p* > 0.05)
								*Post-Fatigue 1*: Peak torque (N.m): CHO: 332.34 ± 48.25 vs PLA: 312.80 ± 47.78	Yes (change from pre-fatigue PLA) ↓ (*p* ≤ 0.05)
								*Post-Fatigue 2*: Peak torque (N.m): CHO: 333.73 ± 45.75 vs PLA: 318.80 ± 40.64;* Post-Fatigue 3:* Peak torque (N.m): CHO: 332.43 ± 44.94 vs PLA: 318.46 ± 33.64	No (*p* > 0.05)
								*Pre-Fatigue:* Average torque (N.m): CHO: 334.10 ± 35.17 vs PLA: 344.61 ± 38.24	No (*p* > 0.05)
								*Post-Fatigue 1:* Average torque (N.m): CHO: 298.11 ± 48.90 vs PLA: 318.71 ± 47.58	Yes (change from pre-fatigue PLA) ↓ (*p* ≤ 0.05)
								*Post-Fatigue 2:* Average torque (N.m): CHO: 301.58 ± 37.58 vs PLA: 317.42 ± 45.19; *Post-Fatigue 3*: Average torque (N.m): CHO: 303.54 ± 34.06 vs PLA: 316.42 ± 43.19	No (*p* > 0.05)
Lane et al. [32]	Cycling TT	12 (males)	Competitive endurance-trained cyclists or triathletes	Overnight	10	10% Maltodextrin with stock solution (contains artificial sweetener and flavouring)	~ 7 days	*Fasted:* Mean mower output (W): CHO: 282 ± 20.78 vs PLA: 273 ± 20.78; *Fed:* Mean power output (W): CHO: 286 ± 20.78 vs PLA: 281 ± 17.32	Yes ↑ (*p* ≤ 0.05)
Phillips et al. [80]	Cycling TT	12 (males)	Active males	2	5	6% Maltodextrin and berry flavouring	3–7 days	Peak power output (W): CHO: 13.51 ± 2.19 vs PLA: 13.20 ± 2.14	Yes ↑ (*p* ≤ 0.05)
								Mean power output (W): CHO: 8.74 ± 0.66 vs PLA: 8.78 ± 0.65	No (*p* > 0.05)
Přibyslavská et al. [81]	Various exercises	11 (female)	NCAA division II Soccer players	Overnight	10–15	6% Maltodextrin and Powerade Zero	7 days	*Jump 1:* Maximum countermovement vertical jump (cm): CHO: 47.3 ± 3.4 vs PLA: 47.7 ± 3.5; *Jump 2*: Maximum countermovement vertical jump (cm): CHO: 48.0 ± 4.1 vs PLA: 47.9 ± 3.5; *Jump 3:* Maximum countermovement vertical jump (cm): CHO: 47.4 ± 3.9 vs PLA: 48.1 ± 3.9	No (*p* > 0.05)
								Mean countermovement vertical jump (cm): CHO: 46.5 ± 3.6 vs PLA: 47.0 ± 3.6; four consutive vertical jumps (cm): CHO: 41.4 ± 3.0 vs PLA: 41.4 ± 3.0; mean 18-m sprint time (s): CHO: 2.87 ± 0.07 vs PLA: 2.86 ± 0.09; mean shuttle run time (s): 16.63 ± 0.34 vs 16.71 ± 0.46	No (*p* > 0.05)
Rollo et al. [82]	Running sprints	11 (males)	Amateur soccer players	**	10	10% Maltodextrin and sweetener	7 days	Mean jogging speed (km·h^−1^): CHO: 11.3 ± 0.7 vs PLA: 10.5 ± 1.3; 15-m sprint distance (km): CHO: 0.16 ± 0.10 vs PLA: 0.15 ± 0.12	Yes ↑ (*p* ≤ 0.05)
								15-m sprint time (s): CHO: 2.65 ± 0.13 vs PLA: 2.69 ± 0.18	No (*p* = 0.298)
Rossato et al. [83]	Running TTE	10 (males)	Recreationally active runners	Overnight (~ 8)	10	6% Maltodextrin and non-caloric juice	48 h	Time (s): CHO: 195.1 ± 51.8 vs PLA: 193.9 ± 46.5	No (*p* = 0.90)
Simpson et al. [84]	Cycling sprints	7 (males)	Recreationally competitive athletes	Overnight	10	6.4% Maltodextrin and Powerade Zero	Min 48 h, max 2 weeks	Mean power (W/kg): CHO: 10.51 ± 0.82 vs PLA: 10.33 ± 0.87	Yes ↑ (*p* = 0.02)
Sinclair et al. [85]	Cycling TT	11 (males)	Recreational cyclists	**	5 & 10	6.4% Maltodextrin	7 days	Distance (km): CHO 10-s rinse: 20.4 ± 2.3 vs PLA: 19.2 ± 2.2	Yes ↑ (*p* ≤ 0.05)
								Mean speed (km/h^−1^): CHO 5-s rinse: 37.95 ± 3.95 vs CHO 10-s rinse: 38.66 ± 4.13 vs PLA: 36.06 ± 4.40; power output (W): CHO 5-s rinse: 152.35 ± 17.42 vs CHO 10-s rinse: 155.63 ± 17.05 vs PLA: 145.73 ± 13.55	Yes, with CHO 10 s vs PLA only ↑ (*p* ≤ 0.05)
Whitham and McKinney [86]	Running TT	7 (males)	Recreationally active	4	5	6% Maltodextrin and lemon juice	Min 5 days	Mean distance (m): CHO: 9333 ± 988 vs PLA: 9309 ± 993	No (*p* = 0.933)

*CHO* carbohydrate oral rinse, *CON* control, *PLA* placebo rinse, *TTE* time to exhaustion, *TT* time trial, *WAT* water rinse

**Not reported or specified

↑Indicates that the statistically significant change is an increase in the performance outcome

↓Indicates that the statistically significant change is a decrease in the performance outcome. Where possible, a specific *p*-value was included for each study

#### Conventional Meta-Analytic Method—Standardised Mean Differences (SMD)

The effects of oral rinsing were analysed in terms of means and standard deviations comparing CHO and placebo (PLA) treatments at the end of the study time. Thus, the standardised mean difference (SMD) was calculated using the Hedges’ *g* method for each individual effect (CHO vs PLA) reported in each study. The Hedges’ *g* method was adjusted using exact computation for the bias-correction factor and Hedges and Oikin were used for standard error for each individual effect size. Conventional meta-analytic techniques rely on the assumption that effect size estimates from different studies are independent and have sampling distributions with known conditional variances [66, 67]. Initially, a conventional random effects (restricted maximum likelihood) meta-analysis model was used to compare the carbohydrate-based oral rinse and placebo rinse conditions. However, this conventional approach was unable to account for the multiple dependent effect sizes (SMDs) from each article included within the current review.

#### Robust Variance Estimation (RVE) Meta-Regression

Subsequently, a random effects meta-regression model using robust variance estimation [67] was used to compare the overall effect of a carbohydrate-based oral rinse on exercise performance. This model technique provides a robust method for estimating standard errors in a meta-regression, particularly when there are dependent effects as was the case in this review. This meta-regression technique was used as a constant-only model and compared with the conventional pairwise meta-analytic method used earlier.

#### Moderators

To explore the effects of moderators, a subgroup analysis was performed on the following moderators: (1) participant characteristics; (2) oral rinse protocols; (3) exercise protocol (i.e. cycling, running etc.) and (4) fasting. Heterogeneity was assessed initially using the *I*^2^ statistic and a value over 50% was deemed to represent substantial heterogeneity [60].

#### Conventional Subgroup Meta-Analysis and a More Robust Variance Approach

Similar issues of dependence within subgroups arose, so initially, conventional techniques to explore the potential statistical heterogeneity of the subgroups were used. Random effects meta-regression models using robust variance estimation were then applied to explore the moderators separately. A sensitivity analysis for categorical moderator variables was conducted for groups with less than four studies, by removing the group from the analysis. With fewer than four studies, the small sample adjustments are deemed inaccurate and hence could not be done with this modelling approach [67].

All analyses were performed through the statistical analysis software Stata (StataCorp 2019. *Stata Statistical Software: Release 16*. College Station, TX, USA: StataCorp LLC), with a significance level set at 5% (*p* < 0.05). Stata meta-analysis packages used were: *meta* (for conventional pairwise and subgroup meta-analysis) and *robumeta* (random effects meta-regression model using robust variance estimation) [67] (https://onlinelibrary.wiley.com/doi/abs/10.1002/jrsm.1091).

## Results

### Systematic Review Results

#### Search Results

In total, 35 studies were eligible for inclusion in the systematic review and 34 studies were eligible for inclusion in the meta-analysis. The main results and characteristics from the included studies are displayed in Table 1.

#### Characterisation of Participants

Across the studies, the sample size ranged from seven to 21 participants, totalling 444 participants. Of these 444 participants, 380 were males and 64 were females. Of the total 35 studies, the majority of included studies recruited only male participants (*n* = 28), while only two studies recruited female participants. The remaining studies recruited both males and females (*n* = 5).

#### Oral Rinse Protocols

Between the studies, there was a large variation in oral rinse protocol. The duration of rinsing ranged from five to 40 s. From the total 35 studies, the majority of studies rinsed for either five (*n* = 12) or 10 s (*n* = 20). The majority used a concentration of 6.4% (*n* = 19); however, across the studies, the concentrations used varied between 6% (*n* = 9), 6.5% (*n* = 1), 8% (*n* = 2), 10% (*n* = 3), 12% (*n* = 1) and 18% (*n* = 1). Further information is shown in Table 1.

#### Origins of Maltodextrin

Twenty-two studies provided information on the type of maltodextrin or the manufacturer that was used in the research. Of all 35 studies, only a small number provided specific information on the composition of the maltodextrin (*n* = 3). Refer to Table 2 for a detailed list of this information. As highlighted previously, information concerning the origin or structure of the maltodextrin should be discussed. These are potentially confounding factors that could affect the efficacy of the carbohydrate oral rinse and any associated exercise performance improvements.

**Table 2:** Origins of maltodextrin used in the included studies

Study	Origin of maltodextrin
Andersson et al. [39]	MyProtein, Northwich, UK
Ataide-Silva et al. [29]	Neonutri-Malto, CHO
Bailey et al. [68]	NS
Bastos-Silva et al. [69]	NS
Bavaresco Gambassi et al. [30]	Athletica Nutrition, Matao, SP, Brazil
Bazzucchi et al. [2]	NS
Beelen et al. [40]	AVEBE (Veendam, The Netherlands)
Black et al. [70]	L.D. Carlson Co., Kent, OH, USA
Carter et al. [28]	NS
Chambers et al. [19]	Roquette, France
Cherif et al. [41]	SIS company, Nelson, UK
Chong et al. [42]	Polycose, Ross Laboratory, Columbus OH
Clarke et al. [71]	MyProtein, Manchester, UK
Clarke et al. [72]	MyProtein, Manchester, UK
Clarke et al. [73]	MyProtein, Cheshire, England, UK
Cramer et al. [74]	NS
de Oliveira et al. [75]	NS
Decimoni et al. [38]	Body Action, Brazil
Deighton et al. [76]	NS
Dorling and Earnest [43]	HighFive, Bardon, England
Dunkin and Phillips [44]	Bulk Powders TM, Colchester, UK
Durkin et al. [77]	NS
Fares and Kayser [31]	NS
Gam et al. [45]	Polycose, Ross Nutrition, Columbus, OH
Green et al. [78]	Natural Foods Inc., Toledo, OH
Jeffers et al. [46]	NS
Jensen et al. [79]	NS
Lane et al. [32]	NS
Phillips et al. [80]	HighFive, Bardon, Leicestershire
Přibyslavská et al. [81]	Letco Medical, Decatur, AL
Rollo et al. [82]	MuscleTalk, Northhamptonshire, UK
Rossato et al. [83]	Health Labs, Belo Horizonte, Brazil
Simpson et al. [84]	Home Brew Supply LLC, TX, USA
Sinclair et al. [85]	NS
Whitham and McKinney [86]	97% polysaccharide, 2% disaccharide, 1% glucose; Roquette, Corby, UK

*NS* not stated

#### Exercise Protocol

The most common type of exercise protocol used in the included studies was a cycling time trial (*n* = 10). Time to exhaustion (TTE) cycling test protocol (*n* = 2) and a cycling sprinting protocol (*n* = 2) were also used. Multiple studies used protocols involving running-based sprinting (*n* = 4), while studies also used protocols involving a running time trial (*n* = 2) and a TTE running test (*n* = 1). The remaining studies included resistance exercise (*n* = 6), isometric contractions (*n* = 2), various exercises (i.e. 10-m running-based sprint, countermovement jump height, isometric mid-thigh pull peak force, repetitions [bench press and squat]) (*n* = 2), walking (*n* = 1), arm cranking (*n* = 1), isometric knee flexion (*n* = 1) and maximum voluntary contractions (*n* = 1).

#### Performance Outcomes

From the total of 35 studies that used a maltodextrin-based oral rinse, a proportion of these studies found a statistically significant improvement in exercise performance (*n* = 19), while the remaining studies found no significant improvements (*n* = 16). Across the studies, the most frequently reported performance outcomes were specific to time (including time to completion and time to exhaustion) (*n* = 21), power (*n* = 12), repetitions (*n* = 7), distance (*n* = 5) and countermovement jumps (*n* = 4). Additional performance outcomes included outcomes specific to speed (*n* = 3), maximal voluntary contractions (*n* = 3), total exercise volume (*n* = 3), work (*n* = 2), training load (*n* = 2), pull peak force (*n* = 1), motor evoked potential (*n* = 1) and torque (*n* = 1). Table 1 presents further information concerning performance outcomes, participant training status and exercise protocol.

#### Fasting

Of the 35 included studies, the majority of studies incorporated fasting into their protocol (*n* = 24) while the remaining studies did not specify if their participants exercised in a fed or fasted state (*n* = 11). Fasting times ranged from 90 min (*n* = 1) to fasting overnight (approximately 8–12 h) (*n* = 16). For a small portion of the studies, the fasting duration was < 5 h (*n* = 8), while for the majority of studies, the fasting duration was > 5 h (*n* = 16).

#### Washout Period

Of the 35 studies, the majority (*n* = 34) included information about a washout period in between trials. One study did not include information concerning washout periods and the author was contacted for further information, with no response received. The shortest reported washout period was 24 h (*n* = 1) and the longest washout period was 7–9 days (*n* = 1). The most common washout period was 7 days (*n* = 9). Table 1 presents more detailed information.

#### Quality Assessment

All studies clearly reported the intervention and outcomes of their research. Of the 35 included studies, only eight sufficiently reported inclusion and exclusion criteria for their participant groups. Thirty-four studies reported participant demographic information and all studies (*n* = 35) included a representative population. The majority of studies (*n* = 34) sufficiently described the methods and protocol used throughout data collection. Twenty-three of the studies described the setting of data collection (e.g. a laboratory-based setting). Most studies disclosed information concerning randomisation (*n* = 28), double blinding (*n* = 24) or single blinding (*n* = 34) in their methodology. The majority of studies (*n* = 29) were based on valid research methods and referenced the original research. All studies (*n* = 35) used consistent measurements and discussed the findings of their research. None of the studies stated participant withdrawals hence no reasons for withdrawals were stated. Seventeen studies discussed biases or limitations of their research. Any potential conflicts of interest were disclosed by 19 of the studies and one paper partially did so. Results of the methodological quality assessment of the studies is presented in Online Resource 1 (see Electronic Supplementary Material [ESM]).

### Meta-Analysis Results

The initial conventional pairwise meta-analysis results showed that the carbohydrate oral rinse increased exercise performance significantly when compared with the placebo rinse condition (SMD = 0.15, 95% CI 0.04, 0.27; *p* = 0.01). However, from this analysis, moderate heterogeneity was observed (*I*^2^ = 20.70%, Tau^2^ = 0.04, Chi^2^ = 83.92, *df* = 57, *p* = 0.01) and as this approach assumes independence between each data point, this approach was not appropriate. To overcome this issue, a random effect robust meta-analysis model was used to compare the overall effect of carbohydrate rinsing on exercise performance. This more conservative adjusted model demonstrated that there was evidence of the carbohydrate oral rinse improving exercise performance in comparison with a placebo oral rinse (SMD = 0.17, 95% CI − 0.01, 0.34; *p* = 0.051). However, the model adjustment to the standard errors resulted in a wider, more conservative 95% confidence interval and *p*-value (towards the null).

### Subgroup Analysis Results—Conventional Subgroup Meta-Analysis

#### Characterisation of Participants

**Fig. 2:**
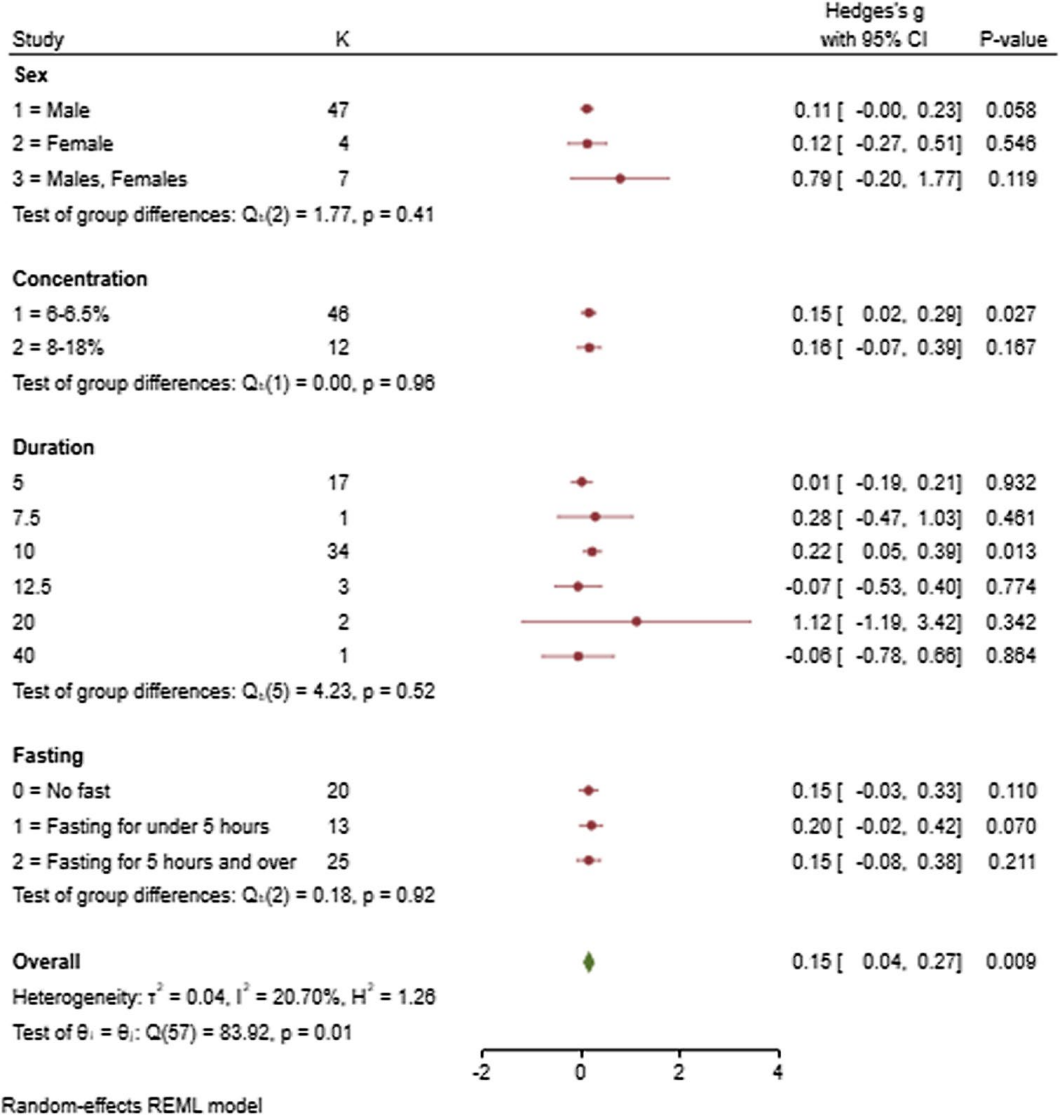
Forest plot comparing the effects of the moderators—sex, rinse concentration (%), rinse duration (s) and fasting—on carbohydrate oral rinsing in comparison with a placebo condition. This forest plot was performed with a conventional random-effects REML (restricted maximum likelihood) model

For the individual groups of male participants (*n* = 47) (SMD = 0.11, 95% CI − 0.004, 0.23; *p* = 0.058), female participants (*n* = 4) (SMD = 0.12, 95% CI − 0.27, 0.51; *p* = 0.546) and combined (male and female participants) (*n* = 7) (SMD = 0.79, 95% CI − 0.20, 1.77; *p* = 0.119), the mean effect size for these groups was not statistically significant at the 5% level (Fig. 2). The combined group showed some unexplained between-study heterogeneity with estimated *I*^2^ of 90.98% within this subgrouping.

#### Oral Rinse Protocols—Rinsing Duration

For the individual groups of rinsing  for 5 s (*n* = 17) (SMD = 0.01, 95% CI − 0.19, 0.21; *p* = 0.932), 7.5 s (*n* = 1) (SMD = 0.28, 95% CI − 0.47, 1.03; *p* = 0.461), 12.5 s (*n* = 3) (SMD = − 0.07, 95% CI − 0.53, 0.40; *p* = 0.774), 20 s (*n* = 2) (SMD = 1.12, 95% CI − 1.19, 3.42; *p* = 0.342) and 40 s (*n* = 1) (SMD = − 0.06, 95% CI − 0.78, 0.66; *p* = 0.864), the mean effect size was not statistically significant (Fig. 2). For the individual group of rinsing for  10 s (*n* = 34) (SMD = 0.22, 95% CI 0.05, 0.39; *p* = 0.013), the mean effect size for this group was statistically significant at the 5% level. For this analysis, articles that provided a range for rinsing duration (e.g. 10–15 s), a middle point between the range was used for the analysis. Furthermore, for articles that provided an approximate rinsing time (i.e. ~ 5 s), a value of 4.9 s was used in the analysis.

#### Oral Rinse Protocols—Rinse Concentration

For the individual groups of rinse concentration of 6–6.5% (group 1) and 8–18% (group 2), the mean effect size for group 1 (*n* = 46) (SMD = 0.15, 95% CI 0.02, 0.29; *p* = 0.027) was statistically significant at the 5% level while the mean effect size for group 2 (*n* = 12) (SMD = 0.16, 95% CI − 0.07, 0.39; *p* = 0.167) was not statistically significant at the 5% level (Fig. 2). The variable of ungrouped, individual rinse concentrations was examined in a continuous format which is available in Online Resource 2 (see ESM).

#### Exercise Protocol

For the individual groups of arm cranking (*n* = 1) (SMD = 0.27, 95% CI − 0.51, 1.04), cycling (*n* = 18) (SMD = 0.07, 95% CI − 0.13, 0.26), isometric contractions (*n* = 4) (SMD = 0.58, 95% CI − 0.49, 1.67), resistance exercise (*n* = 15) (SMD = 0.15, 95% CI − 0.04, 0.35), running (*n* = 17) (SMD = 0.22, 95% CI − 0.12, 0.55), maximum voluntary contractions (*n* = 1) (SMD = − 0.02, 95% CI − 0.77, 0.72) and countermovement vertical jump (*n* = 2) (SMD = 0.01, 95% CI − 0.55, 0.57), the mean effect size for these groups was not statistically significant at the 5% level. The isometric contractions group and running group show some unexplained between-study heterogeneity with estimated *I*^2^ of 86.16% and 64.84%, respectively.

#### Fasting

For the individual groups of no fasting (*n* = 20) (SMD = 0.15, 95% CI − 0.03, 0.33; *p* = 0.110), fasting for < 5 h (*n* = 13) (SMD = 0.20, 95% CI − 0.02, 0.42; *p* = 0.070) and fasting for > 5 h (*n* = 25) (SMD = 0.15, 95% CI − 0.08, 0.38; *p* = 0.211), the mean effect size for these groups was not statistically significant at the 5% level (Fig. 2). The group of fasting for > 5 h shows some unexplained between-study heterogeneity with estimated I^2^ of 71.05%.

### Subgroup Analysis Results—Meta-Regression Model with Robust Variance Estimation

#### Characterisation of Participants

There was no significant difference at the 5% level when comparing the individual group of male participants with female participants (difference between SMDs = 0.17, 95% CI − 2.75, 3.08; *p* = 0.68) or the combination of male and female participants (difference between SMDs = 0.42, 95% CI − 0.98, 1.82; *p* = 0.48). All data points (*n*** = **58) were included in this analysis.

#### Oral Rinse Protocols—Rinsing Duration

There was no significant difference at the 5% level when comparing the individual group of rinsing for 5 s with rinsing for 10 s (difference between SMDs = 0.19, 95% CI − 0.07, 0.45; *p* = 0.15). In a *sensitivity analysis*, only 51 data points were included in the meta-regression analysis as small sample adjustments could not be done with groups with fewer than four.

#### Oral Rinse Protocols—Rinse Concentration

There was no significant difference at the 5% level with group 1 (6–6.5%) compared with group 2 (8–18%) (difference between SMDs = − 0.07, 95% CI − 0.36, 0.22; *p* = 0.58). All data points were included in this analysis.

#### Exercise Protocol

There was no significant difference at the 5% level when comparing the individual group of cycling with isometric contractions (difference between SMDs = 0.52, 95% CI − 1.15, 2.19; *p* = 0.42), resistance exercise (difference between SMDs = 0.09, 95% CI − 0.30, 0.48; *p* = 0.62) or running (difference between SMDs = 0.17, 95% CI − 0.35, 0.69; *p* = 0.49). In a *sensitivity analysis*, only 54 data points were included in the meta-regression analysis as small sample adjustments could not be done with groups with fewer than four.

#### Fasting

There was no significant difference at the 5% level when comparing the individual group of no fasting with fasting group 1 (< 5 h) (difference between SMDs = − 0.04, 95% CI − 0.42, 0.33; *p* = 0.81) or fasting group 2 (> 5 h) (difference between SMDs = − 0.11, 95% CI − 0.55, 0.34; *p* = 0.62). All data points were included in this analysis.

### Risk of Bias Assessment Results

Overall, of the 34 total studies (58 data points) included in this meta-analysis, all demonstrated a high level of evidence. The majority of studies reported using random sequence generation (*n* = 28) and all studies scored a low risk of bias in the categories of incomplete data (*n* = 34) and selective reporting (*n* = 34). A proportion of studies had a single-blinded study design (*n* = 10) and therefore scored an unclear risk of bias for that category. Furthermore, for the category of detection bias, all studies (*n* = 34) scored an unclear risk of bias. Results of the risk of bias assessment of the studies is presented in Online Resource 3 (see ESM).

## Discussion

The purpose of this systematic review and meta-analysis was to investigate the published data examining the effects of a maltodextrin-based, carbohydrate oral rinse on exercise performance. This is the first review to examine the effects of a maltodextrin-based oral rinse on exercise performance and additionally to account for the issue of multiple data points from selected studies when performing the analysis. The key findings from this review were that evidence from the adjusted, random effects meta-regression approach suggests that a maltodextrin-based, carbohydrate oral rinse may cause improvements in exercise performance. Although the conventional meta-analytic approach demonstrated that oral rinsing with a 6–6.5% maltodextrin-based, carbohydrate oral rinse for 10 s was the most effective at improving exercise performance, this approach was deemed to be inaccurate.

The systematic review and meta-analysis published by Brietzke et al. [49] examined the effects of carbohydrate rinsing on cycling time trial performance. Brietzke et al. [49] found that during a cycling time trial, a carbohydrate oral rinse improved mean power output but not the time to completion. However, a limitation exists with the meta-analyses that were performed in this review. In these analyses, multiple data points from three separate studies were included and this was not considered when performing a standard random effects model for the analysis. Similarly, the systematic review and meta-analysis from Pochmuller et al. [51] examined the effects of carbohydrate supplementation on performance trials. Pochmuller et al. [51] found that for trained male cyclists, there may be benefits to ingesting carbohydrates in a concentration of 6–8% prior to and/or during exercise longer than 90 min. To yield more homogeneous study designs, the data was divided into four groups for analysis. However, within these groups, multiple data points from studies were used in the analysis, which was not taken into account or adjusted for. Subsequently, this may have resulted in the analyses being inaccurate or too imprecise.

### Characterisation of Participants

According to the conventional meta-analytic method, there is evidence that the moderator of sex contributed to an improvement in exercise performance. Of the groups in the meta-analysis, only the male participant group was close to reaching significance. The results may be due to confounding factors that were independent of carbohydrate oral rinsing. Differences between males and female participants were not accounted for. However, according to the adjusted, conservative, meta-regression model using robust variance estimation, there was no indication that the moderator of sex contributes to an improvement in exercise performance with carbohydrate oral rinsing. While the majority of studies in this area recruit male participants, they are often classified as physically active or participate in moderate to high levels of activity [2, 29, 30, 39, 40, 43]. Additional research has recruited participants that either train competitively or are experienced in that particular mode of exercise (i.e. running and cycling) [32, 40, 42, 45, 46, 73] and this is a potential reason for variation in the classification of participants recruited in the studies. There are several advantages of recruiting a specific sample group such as trained or experienced runners for exercise testing such as a running time trial. One advantage is that the risk of injury for the participant is lower. For example, novice runners have the highest proportion of injury in comparison with more experienced runners [87–89]. Additionally, trained participants may provide more consistent data as they require fewer familiarisation sessions prior to testing to decrease possible learning effects and to achieve a higher level of reproducibility [90]. This is particularly imperative with exercise tests such as a cycling time trial where self-pacing during the trial is necessary.

### Oral Rinse Protocols—Rinsing Duration

As demonstrated using the conventional meta-analytic method, of the reported rinsing conditions, exposure of the carbohydrate-based oral rinse in the oral cavity for 10 s was statistically significant in improving exercise performance. Conversely, according to the adjusted, conservative, meta-regression model using robust variance estimation, duration of exposure of the rinse in the oral cavity did not significantly improve exercise performance.

The results from the conventional meta-analytic approach is consistent with research undertaken by Sinclair et al. [85] where the results of a 5-s and a 10-s rinse on exercise performance were compared. The authors reported that participants cycled further in the trial with a 10-s rinse in comparison with the 5-s rinse trial. These findings suggest a duration dose response for the carbohydrate oral rinse. Studies that used longer rinse times of 20 s [68] and 60 s [91] did find improvements in exercise performance compared with a placebo rinse. However, it has been suggested that longer rinse times may cause loss of attention and focus while exercising, which can result in transient declines in power output [45] and can also affect performance through the impairment of breathing entrainment, which is defined as the rhythm of breathing synchronised with the rhythm of exercise [45]. Such a response could cause a reduction of oxygen uptake for a certain workload [92, 93] and also may allow for cyclists to reduce the energy costs of exercise [93]. A limitation for this current conventional meta-analysis approach was that the groups of 7.5 s, 20 s and 40 s had two or fewer studies in the subgroup analyses, which can underpower the analysis.

### Oral Rinse Protocols—Rinse Concentration

The results from the conventional meta-analysis show that when the data was divided into two groups (6–6.5% and 8–18%), of the two groups, only the concentration range of 6–6.5% was found to be statistically significant. It is possible that as the concentration of the rinses increases, it reaches a point where little to no improvements to exercise performance are evident, given that in studies using higher concentrations (10% [41], 12% [73] and 18% [44]), no improvements in exercise performance were evident. Further, James et al. [21] reported performance improvements with a 7% rinse; however, these results did not increase when the concentration of the rinse was increased to 14%. However, according to the meta-regression model using robust variance estimation, the moderator of rinse concentration did not significantly improve exercise performance. These inconsistent findings suggest that perhaps a combination of factors (not purely rinse time or rinse concentration) may elicit a performance improvement in exercise. In contrast, research by Jensen et al. [79], Lane et al. [32] and Rollo et al. [82] used 8%, 10% and 10% rinses, respectively, and did find significant improvements in exercise performance with a carbohydrate oral rinse in comparison with a placebo rinse. A limitation for this current conventional meta-analysis approach was that the group of 12% rinse concentration had one study in the subgroup analysis, which can underpower the analysis.

### Exercise Protocol

The results from the conventional meta-analytic approach demonstrate that the moderator of exercise protocol did not significantly contribute to an exercise performance improvement with a carbohydrate oral rinse. However, the specific exercise protocol of cycling, resistance exercise and running demonstrated some evidence of a performance improvement and the inclusion of further original, independent studies in the current analysis may have resulted in a significant finding. Furthermore, according to the adjusted, conservative, meta-regression model using robust variance estimation, the moderator of exercise protocol did not significantly impact exercise performance improvement with a carbohydrate oral rinse.

Across the literature, improvements in both power output and performance time, associated with a carbohydrate oral rinse, have been reported in various forms of exercise including cycling [19, 28–33]. In cycling-based studies, time trials [19, 29, 32, 40, 45, 46, 80], sprints [42, 84] and time to exhaustion tests [30, 31] on a stationary cycle ergometer were used. A potential explanation for the observed improvements in cycling performance is the inclusion of familiarisation sessions in some studies prior to commencing experimental testing [19, 28–30, 32, 40, 42, 45, 80, 84, 85]. The inclusion of familiarisation trials can increase reliability and reduce the likelihood of learning effects [90], implications that are particularly relevant to exercise tests that require self-pacing by participants (e.g. time trials). Across the studies included in this review, a small selection of studies did not include information on familiarisation protocol [31, 38, 46, 69, 74, 77]. It is currently recommended that for experienced participants, at least one familiarisation trial is required for reproducibility of performance [94–97] and to establish a stable pacing strategy [98].

Similarly, exercise performance improvements with carbohydrate oral rinsing have also been evident in running [34–37]. In running-based studies, the mode of exercise included sprints [41, 43, 82] and time trials [73, 86]. Unlike cycling-based exercise tests, where tests are typically conducted on a cycle ergometer, research methods in running-based studies were mixed, using stationary treadmill running [41, 83, 86] and self-paced running indoors [43, 72, 75, 81, 82] and outdoors [73]. Such variation in exercise protocols can affect overall validity and reliability, and increase variability [99]. For example, laboratory-based running protocols typically have a decreased level of ecological validity [100]. In comparison with self-paced running outside, the decision to consciously control the speed of the treadmill does not occur as often or as swiftly [101, 102]. Small but significant differences also exist between running on a treadmill and running on a track including variation in movement patterns, ground surface and airstream [103].

Improvements in exercise performance associated with carbohydrate oral rinsing have also been found in resistance exercise protocols [38, 77, 78]. In resistance exercise-based studies, the specific exercises included leg press, bench press, military press, seated row and half-squat [38] and bench press protocols [44, 78]. Oral rinsing with maltodextrin has also resulted in improvements in total workload volume during resistance-based exercises in comparison with a placebo [38, 77]. However, this is in comparison with other research using resistance-based exercise where no performance improvements were evident with a carbohydrate oral rinse [44].

Additionally, improvements in exercise performance associated with carbohydrate oral rinsing have also been demonstrated in protocols incorporating isometric contractions [68] and isometric knee extension [79]. Using isometric knee extension, Jensen et al. [79] reported a significant decline in torque with a maltodextrin oral rinse in comparison with a placebo. Bailey et al. [68] demonstrated increases in maximal voluntary contractions with a maltodextrin oral rinse in comparison with a placebo. However, a limitation across the existing literature is that the methods used do not quantify effects on functional activities (e.g. squatting) [68]. Functional activities and training are more representative of daily movement and general exercise movement patterns [104]. One previous study used an arm cranking protocol [39]. However, this study did not find significant differences in distance (km) covered during a 30-min trial with a maltodextrin rinse when compared with a placebo. Furthermore, the aforementioned studies that use resistance exercise, arm cranking, isometric flexion, contraction and extension as modes of exercise to examine the effects of a carbohydrate oral rinse on exercise performance have limited applicability to sport performance more broadly, or specific exercise classifications (e.g. endurance or sprint-based exercise). A limitation for the current conventional meta-analysis approach was that the groups of arm cranking, maximum voluntary contractions and countermovement vertical jump had two or fewer studies in the analysis, which can result in an underpowered analysis for that group.

### Fasting

From the results from the conventional meta-analytic method, it was unclear as to the effect of fasting on exercise performance with a carbohydrate oral rinse. All groups were close to reaching significance. Furthermore, according to the meta-regression model using robust variance estimation, there were no significant effects of fasting on exercise performance while using a carbohydrate oral rinse. These unclear findings are reflected in the literature as it has not been conclusively determined whether exercising in the preprandial or postprandial state contributes to a performance improvement. Fares and Kayser reported improvements in cycling performance time with a maltodextrin-based oral rinse in comparison with a placebo in both preprandial (overnight fasting) and postprandial states [31]. However, this finding of exercise improvements in both the preprandial and prandial state is in contrast with a large amount of literature in the area. For example, Lane et al. [32] found greater power output during a cycling time trial with a maltodextrin oral rinse (in comparison with a placebo) in a fasted state compared with a fed state (3.4% vs 1.8% performance enhancement, respectively). Exercising in a fasted state could possibly be necessary for a carbohydrate-based oral rinse to improve exercise performance. These possible benefits of the carbohydrate oral rinse may therefore be in part dependant on endogenous carbohydrate (liver and glycogen) stores [40]. However, other studies have reported no performance improvements in a fed state in comparison with a fasted state [40, 86]. Beelen et al. [40] reported no improvements in performance time during a cycling time trial in a fed state in comparison with a fasted state and Whitham and McKinney [86] also found no increases in distance ran during a 45-min trial in a fed state in comparison with a fasted state while using a maltodextrin oral rinse in comparison with a placebo. Future research should aim to further investigate the effects of fasting on exercise performance while using a carbohydrate oral rinse.

### Types of Maltodextrin

A limitation of the current research in the area is that the type of maltodextrin each study used in the rinse is often not specified. While most studies do include the manufacturer information of the maltodextrin, few studies include specific information on the composition of the maltodextrin [42, 45, 86]. Two of the studies included in the current review used Polycose (a maltodextrin with a composition of 91% glucose oligomers and polymers, 7% maltose and 2% glucose [105]) [42, 45, 63] and one study used a maltodextrin with a 97% polysaccharide, 2% disaccharide and 1% glucose composition [86]. When such information is provided, further observations can be made which might facilitate further understanding as to how carbohydrate oral rinses improve exercise performance. Without information on the maltodextrin origin (e.g. corn, rice, manioc, oat or potato starch) and structure (amylose and amylopectin ratio, DE and DP), it becomes difficult to make direct comparisons on the performance improvements between studies as the various types of maltodextrin may affect the overall effect of the carbohydrate oral rinse and, in turn, perhaps the findings and results from each study. Future research should aim to specify the composition of the maltodextrins (including information on both DE and origin). Furthermore, more research may be necessary to identify the optimal composition of maltodextrins to trigger the exercise performance increase when using a carbohydrate oral rinse.

### Limitations of Carbohydrate Oral Rinsing

One possible limitation of carbohydrate oral rinsing is the risk of choking on the rinse during exercise; however, no published research has discussed this risk. Furthermore, research by Gam et al. [45] showed that the action of mouth rinsing itself in a cycling time trial may result in impaired exercise performance. Their findings show in a cycling time trial that a water-based placebo rinse resulted in a 2.7% increase in completion time compared with the no-rinse control. They hypothesise that rinsing may cause a loss of attention and ability to focus on the task while also impairing breathing entrainment, which therefore resulted in transient declines in power output [45]. However, the addition of carbohydrates to the mouth rinse was found to oppose this decrease in performance associated with rinsing and may provide additional benefits as opposed to not rinsing at all [45]. These findings contrast, however, with another study that reported similar results with a water-based placebo rinse and a no-rinse control [106]. This study by Gam et al. [45] is the first to discuss limitations associated with carbohydrate oral rinsing itself.

### Meta-Analysis Statistical Approach

As previously discussed, the conventional meta-analytic method used in the current review is often used within previously published systematic reviews and meta-analyses [49, 51]. However, the conventional approach does not account for the multiple dependent effect sizes (SMDs) from each study. Therefore an adjusted approach using a random effects meta-regression model using robust variance estimation was also used in this current review [67]. As this approach is more conservative, many statistically significant results from the conventional approach were no longer significant at the 5% level and 95% confidence intervals crossed zero. It is possible that the moderators included in this review (i.e. sex, oral rinse protocols, exercise protocol, fasting) are potentially associated with the variation of the effect size (SMDs) between studies based on conventional unadjusted results. However, when adjusted using the more conservative approach, results are non-significant. Thus, further independent studies in this area of research may suggest that some of the potential moderators are significantly associated with the variation of the study effect sizes.

### Strength and Limitations

This is the first systematic review and meta-analysis to include all performance-based exercise outcomes and also to include only studies that that used a maltodextrin-based, carbohydrate oral rinse. Another strength of the current review is that five databases were systematically searched to ensure all relevant articles were screened and three independent reviewers were used for the screening process to minimise bias. Further strengths are the large pool of studies included for the systematic review (*n* = 35) and meta-analysis (*n* = 34) and that due to the complex data in the meta-analysis, the analytical approach accounted for these complexities and limitations.

Several limitations with the studies included in the current review need to be acknowledged. One issue with the data provided within the included studies was the lack of standardisation of exercise protocol or oral rinse protocol among the study methodology. For example, within the pool of studies that examined the effect of carbohydrate oral rising on running performance, the protocol varied widely. The exercise protocols included sprints, time trials and time to exhaustion trials; the carbohydrate oral rinse concentration ranged from 6 to 10% and the duration of rinsing ranged from 5 to 40 s. This variability makes it difficult to fully understand the mechanisms that may affect exercise performance. It would be beneficial for future research to follow existing, validated exercise protocols to explore the effects of carbohydrate oral rinsing on exercise performance. A further limitation is the complexity of the data set included in the meta-analysis. Synthesising and analysing the data was difficult due to a wide range of exercise protocols, carbohydrate oral rinsing protocols and methodology across the included literature as well as multiple data points in the data set. However, a unique statistical approach was used to account for these issues, in order to minimise caution with interpretation. A further possible limitation to consider is that the *p*-value for the adjusted, conservative, random effects meta-regression model was on the cusp of achieving significance at the 5% level, with the confidence interval (CI) crossing zero. Consequently, this may indicate the possibility of a higher chance of a type I error.

### Future Research

In this field of research, there are areas that remain largely unknown. These areas include the specific effect of maltodextrin structure and origin on maltodextrin-based carbohydrate oral rinsing; the exact mechanism that facilitates improvements in exercise performance after carbohydrate oral rinsing and the precise receptor systems that are involved in this process. Future research investigating carbohydrate oral rinsing and exercise performance should aim to explore these areas further.

Furthermore, one area that has not been considered in previous literature is the participants’ individual taste sensitivity to complex carbohydrates. Research has demonstrated that individual taste sensitivity to carbohydrates exists [105, 107–109] and that individual sensitivity to complex carbohydrates can vary largely among individuals [109]. From this research, the question emerges as to whether individual taste sensitivity influences the efficacy of a carbohydrate oral rinse on improving exercise performance. Furthermore, if sensitivity does affect this interaction, what is the degree of effect?

Potentially, individual taste sensitivity may moderate the efficacy of a carbohydrate oral rinse and its effect on improving exercise performance. Research has demonstrated that particular groups of people may experience varying outcomes depending on their levels of carbohydrate taste sensitivity. For example, those who experienced high intensity ratings for complex carbohydrates had larger waist circumferences and a significantly higher daily energy intake in comparison with those who experienced a low intensity [107]. Low et al. [110] also demonstrated that carbohydrate hypersensitive participants consumed 50% more maltodextrin-based milkshake in comparison with those who were less sensitive and this was independent to their liking of the beverage. These examples illustrate that oral sensitivity and exposure to complex carbohydrates can affect multiple dimensions including consumption and anthropometry. If an individual’s sensitivity to complex carbohydrates does influence the magnitude of effect a carbohydrate oral rinse has on exercise performance, this may be a cause of variation in each study.

While research exists on the effects of carbohydrate oral rinsing on exercise performance, no existing literature has explored the effect of carbohydrate taste sensitivity on exercise capacity and performance. Future research should explore this area and examine the effect of individual taste sensitivity on the efficacy of a carbohydrate oral rinse and its subsequent effect on exercise performance.

### Practical Applications

Based on the findings of this systematic review and meta-analysis, athletes should consider using a maltodextrin-based, carbohydrate oral rinse to achieve potential improvements in exercise performance.

## Conclusions

Despite conflicting data in the field, this review demonstrates that a maltodextrin-based, carbohydrate oral rinse suggests evidence for improving exercise performance. Although the conventional subgroup analyses demonstrated that a maltodextrin-based, carbohydrate oral rinse was most effective at improving exercise performance when rinsing for 10 s at a concentration between 6 and 6.5%, this meta-analytic approach was considered to be inaccurate. Alternatively, although non-significant, the more robust, adjusted, meta-regression model suggested some evidence of a maltodextrin-based, carbohydrate oral rinse improving exercise performance overall.

## Supplementary Information

Below is the link to the electronic supplementary material.

Supplementary file1 (PDF 239 kb)

Supplementary file2 (PDF 119 kb)

Supplementary file3 (PDF 184 kb)

## Declarations

### Availability of data and material

The datasets generated during and/or analysed during the current study are available from the corresponding author on reasonable request.
