# Supplementary material for: Maltodextrin-Based Carbohydrate Oral Rinsing and Exercise Performance: Systematic Review and Meta-Analysis
Source: Sports Med. 2022 Mar 3;52(8):1833–62. doi: 10.1007/s40279-022-01658-3 (PMC9325805; doi:10.1007/s40279-022-01658-3)
Supplement: Supplementary file 2 — Supplementary file2 (PDF 119 kb) [file 40279_2022_1658_MOESM2_ESM.pdf]

Online Resource 2

Title: Maltodextrin Based, Carbohydrate Oral Rinsing and Exercise Performance: Systematic Review and Meta-Analysis

Journal: Sports Medicine

Authors: Claudia Hartley[1], Amelia Carr[2], Steven J. Bowe[3], Wender L.P. Bredie[4], Russell S.J. Keast[1]

1 CASS Food Research Centre, Deakin University, Burwood Highway, Burwood, VIC 3125, Australia; c.hartley@deakin.edu.au (C.H.); russell.keast@deakin.edu.au (R.S.J.K.)

2 Centre for Sport Research, Deakin University, Burwood Highway, Burwood, VIC 3125, Australia; amelia.carr@deakin.edu.au (A.C.)

3 Deakin Biostatistics Unit, Faculty of Health, Deakin University, Burwood Highway, Burwood, VIC 3125, Australia; s.bowe@deakin.edu.au (S.J.B.)

4 Department of Food Science, University of Copenhagen, Rolighedsvej 26, 1958 Frederiksberg C; wb@food.ku.dk (W.L.P.B.)

Correspondence:
Professor Russell SJ Keast
Email: russell.keast@deakin.edu.au

Online Resource 2: Subgroup analysis results of oral rinse protocols – rinse concentration.

This Online Resource details further statistical analysis performed for the meta-analysis section of the systematic review and meta-analysis.

Subgroup Analysis Results - Conventional Subgroup Meta-Analysis

For the individual groups of rinse concentration of 6% (n = 18) (SMD = 0.17, 95% CI: [-0.09, 0.43], p = 0.205), 6.4% (n = 26) (SMD = 0.15, 95% CI: [-0.03, 0.32], p = 0.093), 6.5% (n = 2) (SMD = 0.14, 95% CI: [-0.52, 0.80], p = 0.677), 8% (n = 2) (SMD = -0.18, 95% CI: [-0.72, 0.36], p = 0.516), 10% (n = 6) (SMD = 0.13, 95% CI: [-0.18, 0.45], p = 0.402) and 18% (n = 3) (SMD = 0.11, 95% CI: [-0.34, 0.55], 0.639), the mean effect size for these groups was not statistically significant at the 5% level. For the individual group of 12% (n = 1) (SMD = 1.14, 95% CI: [0.38, 1.89], p = 0.003) the mean effect size for this group was statistically significant at the 5% level. However this group had one study in the subgroup analysis which may have resulted in an inaccurate result. The 6% and 6.4% groups show some unexplained between-study heterogeneity with estimated I^2 of 51.41% and 20.59% respectively.

Subgroup Analysis Results - Meta-Regression Model with Robust Variance Estimation

There was no significant difference at the 5% level when comparing the individual group of rinse concentration of 6% with 6.4% (differences between SMDs = -0.16, 95% CI: [-0.78, 0.47], p = 0.60) or 10% (difference between SMDs = -0.16, 95% CI: [-1.00, 0.68], p = 0.61). In a *sensitivity analysis*, only fifty data points were included in the meta-regression analysis as small sample adjustments could not be done with groups with less than four.
