# Supplementary material for: Maltodextrin-Based Carbohydrate Oral Rinsing and Exercise Performance: Systematic Review and Meta-Analysis
Source: Sports Med. 2022 Mar 3;52(8):1833–62. doi: 10.1007/s40279-022-01658-3 (PMC9325805; doi:10.1007/s40279-022-01658-3)
Supplement: Supplementary file 3 — Supplementary file3 (PDF 184 kb) [file 40279_2022_1658_MOESM3_ESM.pdf]

Online Resource 3

Title: Maltodextrin Based, Carbohydrate Oral Rinsing and Exercise Performance: Systematic Review and Meta-Analysis

Journal: Sports Medicine

Authors: Claudia Hartley[1], Amelia Carr[2], Steven J. Bowe[3], Wender L.P. Bredie[4], Russell S.J. Keast[1]

1 CASS Food Research Centre, Deakin University, Burwood Highway, Burwood, VIC 3125, Australia; c.hartley@deakin.edu.au (C.H.); russell.keast@deakin.edu.au (R.S.J.K.)

2 Centre for Sport Research, Deakin University, Burwood Highway, Burwood, VIC 3125, Australia; amelia.carr@deakin.edu.au (A.C.)

3 Deakin Biostatistics Unit, Faculty of Health, Deakin University, Burwood Highway, Burwood, VIC 3125, Australia; s.bowe@deakin.edu.au (S.J.B.)

4 Department of Food Science, University of Copenhagen, Rolighedsvej 26, 1958 Frederiksberg C; wb@food.ku.dk (W.L.P.B.)

Correspondence:

Professor Russell SJ Keast

Email: russell.keast@deakin.edu.au

Online Resource 3: Risk of Bias Assessment of studies included in the meta-analysis.

This Online Resource details the Risk of Bias Assessment that was performed. The studies included were included in the meta-analysis.

	Random sequence generation (selection bias)	Blinding of participants and personnel (performance bias)	Blinding of outcome (detection bias)	Incomplete data (attrition bias)	Selective reporting (reporting bias)
Andersson et al. [39]	+	?	?	+	+
Ataide-Silva et al. [29].	+	+	?	+	+
Bailey et al. [68]	+	+	?	+	+
Bastos-Silva et al. [69]	+	?	?	+	+
Bastos-Silva et al. [69]	+	?	?	+	+
Bavaresco Gambassi et al. [30]	+	+	?	+	+
Bazzucchi et al. [2]	+	+	?	+	+
Beelen et al. [40]	+	+	?	+	+
Beelen et al. [40]	+	+	?	+	+
Black et al. [70]	+	+	?	+	+
Carter et al. [28]	?	?	?	+	+
Carter et al. [28]	?	?	?	+	+
Chambers et al. [19]	+	+	?	+	+
Chambers et al. [19]	+	+	?	+	+
Cherif et al. [41]	+	?	?	+	+
Cherif et al. [41]	+	?	?	+	+
Chong et al. [42]	+	+	?	+	+
Clarke et al. [71]	+	+	?	+	+
Clarke et al. [71]	+	+	?	+	+

Clarke et al. [71]	+	+	?	+	+
Clarke et al. [72]	+	?	?	+	+
Clarke et al. [72]	+	?	?	+	+
Clarke et al. [72]	+	?	?	+	+
Clarke et al. [72]	+	?	?	+	+
Clarke et al. [72]	+	?	?	+	+
Clarke et al. [73]	+	?	?	+	+
Clarke et al. [73]	+	?	?	+	+
Cramer et al. [74]	?	+	?	+	+
Cramer et al. [74]	?	+	?	+	+
de Oliveira et al. [75]	+	+	?	+	+
de Oliveira et al. [75]	+	+	?	+	+
Decimoni et al. [38]	+	+	?	+	+
Dorling, Earnest [43]	+	+	?	+	+
Dorling, Earnest [43]	+	+	?	+	+
Dorling, Earnest [43]	+	+	?	+	+
Dunkin, Phillips [44]	+	+	?	+	+
Dunkin, Phillips [44]	+	+	?	+	+
Dunkin, Phillips [44]	+	+	?	+	+
Durkin et al. [77]	?	?	?	+	+
Durkin et al. [77]	?	?	?	+	+
Durkin et al. [77]	?	?	?	+	+
Fares, Kayser [31]	+	?	?	+	+
Gam et al. [45]	+	?	?	+	+
Green et al. [78]	+	+	?	+	+
Jeffers et al. [46]	+	+	?	+	+
Jensen et al. [79]	+	+	?	+	+
Lane et al. [32]	?	+	?	+	+
Phillips et al. [80]	+	+	?	+	+
Přibyslavská et al. [81]	?	+	?	+	+
Přibyslavská et al. [81]	?	+	?	+	+
Přibyslavská et al. [81]	?	+	?	+	+
Rollo et al. [82]	+	+	?	+	+
Rollo et al. [82]	+	+	?	+	+

Rollo et al. [82]	+	+	?	+	+
Rossato et al. [83]	+	+	?	+	+
Simpson et al. [84]	+	+	?	+	+
Sinclair et al. [85]	?	?	?	+	+
Whitham, McKinney [86]	+	+	?	+	+

Reference List:

2. Bazzucchi I, Patrizio F, Felici F, Nicolò A, Sacchetti M. Carbohydrate Mouth Rinsing: Improved Neuromuscular Performance During Isokinetic Fatiguing Exercise. Int J Sports Physiol Perform. 2017;12(8):1031-8. doi:10.1123/ijspp.2016-0583.

19. Chambers ES, Bridge MW, Jones DA. Carbohydrate sensing in the human mouth: effects on exercise performance and brain activity. The Journal of physiology. 2009;587(Pt 8):1779-94. doi:10.1113/jphysiol.2008.164285.

28. Carter JM, Jeukendrup AE, Jones DA. The effect of carbohydrate mouth rinse on 1-h cycle time trial performance. Med Sci Sports Exerc. 2004;36(12):2107-11. doi:10.1249/01.mss.0000147585.65709.6f.

29. Ataide-Silva T, Ghiarone T, Bertuzzi R, Stathis CG, Leandro CG, Lima-Silva AE. CHO Mouth Rinse Ameliorates Neuromuscular Response with Lower Endogenous CHO Stores. Med Sci Sports Exerc. 2016;48(9):1810-20. doi:10.1249/mss.0000000000000973.

30. Bavaresco Gambassi B, Gomes de Santana Barros Leal Y, Pinheiro Dos Anjos ER, Antonelli BA, Gomes Goncalves ESDC, Hermes Pires de Melo Montenegro I et al. Carbohydrate mouth rinse improves cycling performance carried out until the volitional exhaustion. J Sports Med Phys Fitness. 2019;59(1):1-5. doi:10.23736/s0022-4707.17.07980-4.

31. Fares EJ, Kayser B. Carbohydrate mouth rinse effects on exercise capacity in pre- and postprandial States. J Nutr Metab. 2011;2011:385962. doi:10.1155/2011/385962.

32. Lane SC, Bird SR, Burke LM, Hawley JA. Effect of a carbohydrate mouth rinse on simulated cycling time-trial performance commenced in a fed or fasted state. Appl Physiol Nutr Metab. 2013;38(2):134-9. doi:10.1139/apnm-2012-0300.

39. Andersson H, Knight A, Buscombe R, Sinclair J, Edmonds CJ, Bottoms L. The effect of carbohydrate mouth rinse on a 30-minute arm cranking performance. Comp Exerc Physiol. 2016;12(1):41-7. doi:10.3920/CEP150032.

40. Beelen M, Berghuis J, Bonaparte B, Ballak SB, Jeukendrup AE, van Loon LJ. Carbohydrate mouth rinsing in the fed state: lack of enhancement of time-trial performance. Int J Sport Nutr Exerc Metab. 2009;19(4):400-9. doi:10.1123/ijsnem.19.4.400.

41. Cherif A, Meeusen R, Ryu J, Taylor L, Farooq A, Kammoun K et al. Repeated-sprints exercise in daylight fasting: carbohydrate mouth rinsing does not affect sprint and reaction time performance. Biol Sport. 2018;35(3):237-44. doi:10.5114/biolsport.2018.77824.

42. Chong E, Guelfi KJ, Fournier PA. Effect of a carbohydrate mouth rinse on maximal sprint performance in competitive male cyclists. J Sci Med Sport. 2011;14(2):162-7. doi:10.1016/j.jsams.2010.08.003.

43. Dorling JL, Earnest CP. Effect of carbohydrate mouth rinsing on multiple sprint performance. J Int Soc Sports Nutr. 2013;10(1):41. doi:10.1186/1550-2783-10-41.

44. Dunkin JE, Phillips SM. The Effect of a Carbohydrate Mouth Rinse on Upper-Body Muscular Strength and Endurance. J Strength Cond Res. 2017;31(7):1948-53. doi:10.1519/jsc.0000000000001668.

45. Gam S, Guelfi KJ, Fournier PA. Opposition of Carbohydrate in a Mouth-Rinse Solution to the Detrimental Effect of Mouth Rinsing During Cycling Time Trials. Int J Sport Nutr Exerc Metab. 2013;23(1):48.

46. Jeffers R, Shave R, Ross E, Stevenson EJ, Goodall S. The effect of a carbohydrate mouth-rinse on neuromuscular fatigue following cycling exercise. Appl Physiol Nutr Metab. 2015;40(6):557-64.

68. Bailey SP, Hibbard J, La Forge D, Mitchell M, Roelands B, Harris GK et al. Impact of a Carbohydrate Mouth Rinse on Quadriceps Muscle Function and Corticomotor Excitability. International Journal of Sports Physiology & Performance. 2019;14(7):927-33.

69. Bastos-Silva VJ, Prestes J, Geraldes AAR. Effect of Carbohydrate Mouth Rinse on Training Load Volume in Resistance Exercises. Journal of strength and conditioning research. 2019;33(6):1653-7. doi:10.1519/JSC.0000000000002092.

70. Black CD, Schubert DJ, Szczyglowski MK, Wren JD. Carbohydrate Mouth Rinsing Does Not Prevent the Decline in Maximal Strength After Fatiguing Exercise. Journal of Strength & Conditioning Research. 2018;32(9):2466-73.

71. Clarke ND, Kornilios E, Richardson DL. Carbohydrate and Caffeine Mouth Rinses Do Not Affect Maximum Strength and Muscular Endurance Performance. Journal of strength and conditioning research. 2015;29(10):2926-31. doi:10.1519/JSC.0000000000000945.

72. Clarke ND, Hammond S, Kornilios E, Mundy PD. Carbohydrate mouth rinse improves morning high-intensity exercise performance. European Journal of Sport Science. 2017;17(8):955-63.

73. Clarke ND, Thomas JR, Kagka M, Ramsbottom R, Delextrat A. No Dose-Response Effect of Carbohydrate Mouth Rinse Concentration on 5-km Running Performance in Recreational Athletes. Journal of strength and conditioning research. 2017;31(3):715-20. doi:10.1519/JSC.0000000000001531.

74. Cramer MN, Thompson MW, Périard JD. Thermal and Cardiovascular Strain Mitigate the Potential Benefit of Carbohydrate Mouth Rinse During Self-Paced Exercise in the Heat. Frontiers in physiology. 2015;6:354. doi:10.3389/fphys.2015.00354.

75. de Oliveira JJ, de Souza Salles AH, Reis Barbosa CG, de Oliveira Silva JA, de Melo Dias R, Silva Vilela Ribeiro AG et al. Effect of Post-Activation Potentiation and Carbohydrate Mouth Rise on Repeated Sprint Ability in University Futsal Players. Journal of Exercise Physiology Online. 2020;23(2):29-40.

77. Durkin M, Akeroyd H, Holliday A. Carbohydrate mouth rinse improves resistance exercise capacity in the glycogen-lowered state. Applied Physiology, Nutrition & Metabolism. 2021;46(2):126-32.

78. Green MS, Kimmel CS, Martin TD, Mouser JG, Brune MP. Effect of Carbohydrate Mouth Rinse on Resistance Exercise Performance. Journal of strength and conditioning research. 2020. doi:10.1519/JSC.0000000000003755.

79. Jensen M, Stellingwerff T, Klimstra M. Carbohydrate mouth rinse counters fatigue related strength reduction. International Journal of Sport Nutrition and Exercise Metabolism. 2015;25(3):252-61. doi:10.1123/ijsnem.2014-0061.

80. Phillips SM, Findlay S, Kavaliauskas M, Grant MC. The influence of serial carbohydrate mouth rinsing on power output during a cycle sprint. Journal of Sports Science and Medicine. 2014;13(2):252-8.

81. Přibyslavská V, Scudamore EM, Johnson SL, Green JM, Stevenson Wilcoxson MC, Lowe JB et al. Influence of carbohydrate mouth rinsing on running and jumping performance during early morning soccer scrimmaging. European journal of sport science. 2016;16(4):441-7. doi:10.1080/17461391.2015.1020345.

82. Rollo I, Homewood G, Williams C, Carter J, Goosey-Tolfrey VL. The Influence of Carbohydrate Mouth Rinse on Self-Selected Intermittent Running Performance. Int J Sport Nutr Exerc Metab. 2015;25(6):550.

83. Rossato LT, Fernandes CTM, Vieira PF, de Branco FMS, Nahas PC, Puga GM et al. No Improvement in Running Time to Exhaustion at 100% VO2max in Recreationally Active Male Runners With a Preexercise Single-Carbohydrate Mouth Rinse. International journal of sports physiology and performance. 2019:1178-83. doi:10.1123/ijspp.2018-0624.

84. Simpson GW, Pritchett R, O'Neal E, Hoskins G, Pritchett K. Carbohydrate Mouth Rinse Improves Relative Mean Power During Multiple Sprint Performance. International journal of exercise science. 2018;11(6):754-63.

85. Sinclair J, Bottoms L, Flynn C, Bradley E, Alexander G, McCullagh S et al. The effect of different durations of carbohydrate mouth rinse on cycling performance. European journal of sport science. 2014;14(3):259-64. doi:10.1080/17461391.2013.785599.

86. Whitham M, McKinney J. Effect of a carbohydrate mouthwash on running time-trial performance. Journal of Sports Sciences. London; UK: Taylor & Francis; 2007. p. 1385-92.
